# Inpatient Point-of-Care HIV Early Infant Diagnosis in Mozambique to Improve Case Identification and Linkage to Antiretroviral Therapy

**DOI:** 10.9745/GHSP-D-20-00611

**Published:** 2021-03-31

**Authors:** Mércia Matsinhe, Timothy Bollinger, Nilza Lee, Osvaldo Loquiha, Bindiya Meggi, Nédio Mabunda, Chishamiso Mudenyanga, Dadirayi Mutsaka, Marcelina Florêncio, Aurora Mucaringua, Eugénia Macassa, Amir Seni, Ilesh Jani, W. Chris Buck

**Affiliations:** a Hospital Central de Maputo, Maputo, Mozambique.; b Instituto Nacional de Saúde, Maputo, Mozambique.; c Clinton Health Access Initiative, Maputo, Mozambique.; d Hospital Central de Beira, Beira, Mozambique.; e University of California Los Angeles, David Geffen School of Medicine, Los Angeles, USA.

## Abstract

**Introduction:**

Novel approaches to case identification and linkage to antiretroviral therapy (ART) are needed to close gaps in early infant diagnosis (EID) of HIV. Point-of-care (POC) EID is a recent innovation that eliminates the long turnaround times of conventional EID that limit patient management in the inpatient setting. The initial deployment of POC EID in Mozambique focused primarily on outpatient clinics; however, 2 high-volume tier-4 pediatric referral hospitals were also included.

**Methods:**

To assess the impact of inpatient POC EID, a retrospective review of testing and care data from Hospital Central de Beira (HCB) and Hospital Central de Maputo (HCM) was performed for the period September 2017 to July 2018, with comparison to the 8-month pre-POC period when dried blood spots were used for conventional EID.

**Results:**

Monthly testing volume increased from 8.5 tests pre-POC to 17.6 tests with POC (*P*<.001). Among 511 children with POC testing, the median age was 5 months, there was ongoing breastfeeding in 326 (63.8%), and 136 (26.6%) of mothers and 146 (28.6%) of infants had not received ART or antiretroviral prophylaxis, respectively. POC tests were positive in 152 (29.7%) infants, and 52 (37.5%) had a previous negative DNA polymerase chain reaction through the conventional outpatient EID program. Linkage to ART for infants with HIV-positive tests improved 64% during the POC period (*P*=.002). Inpatient mortality for infected infants during the POC period was 28.2%. Excluding these deaths, 61.2% of eligible infants initiated ART as inpatients, but only 29.8% of those discharged without ART were confirmed to have initiated as outpatients.

**Conclusions:**

Inpatient wards are a high-yield site for EID and ART initiation that have historically been overlooked in programming for prevention of mother-to-child transmission. POC platforms represent a transformative opportunity to increase inpatient testing, make definitive diagnoses, and improve timely linkage to ART. Scale-up plans should prioritize pediatric wards.

## Abstract (precis)

Introduction of point-of-care early infant diagnosis on the inpatient wards of 2 of the largest pediatric referral hospitals in Mozambique increased HIV testing volume and pediatric HIV case identification with improved linkage to antiretroviral therapy.

## Teaser

**Key Findings:**

- Monthly virologic testing volume in HIV-exposed infants increased 97% with introduction of point-of-care early infant diagnosis testing.
- There was a 29.7% positivity rate for inpatient point-of-care tests performed.
- Antiretroviral therapy initiation for infants with positive tests improved 64% with introduction of point-of-care early infant diagnosis.

**Key Implications:**

- Pediatric inpatient wards are high-yield sites for HIV case identification, and inpatient early infant diagnosis is an important backstop to outpatient follow-up of exposed infants for prevention of mother-to-child transmission.
- Point-of-care testing improves inpatient early infant diagnosis performance, facilitating more timely initiation of antiretroviral in HIV-infected infants, and point-of-care early infant diagnosis expansion plans should prioritize high-volume pediatric wards.

## INTRODUCTION

Despite massive scale-up over the past decade, pediatric antiretroviral therapy (ART) coverage rates in sub-Saharan Africa remain low. In Mozambique, only an estimated 50% of HIV-infected children were on ART at the end of 2018, compared with a 55% coverage rate in adults.^1^^,^^2^ Low pediatric coverage rates can, in part, be attributed to significant challenges with retention of mother–infant pairs in prevention of mother-to-child transmission (PMTCT) services with only 62% of exposed infants of women living with HIV enrolled in antenatal care having an early infant diagnosis (EID) virologic test by 2 months of age in 2018.^1^ For HIV-exposed infants (HEI) retained in care, the complexity of establishing the HIV status of children under 18 months of age likely also contributes to low pediatric ART coverage. Definitive diagnosis in HEIs requires a virologic EID test, which until recently was only available in centralized reference laboratories, with delayed result delivery due to transport times and the need to process samples from many health facilities.^3^ Slow delivery of EID results and subsequent delayed ART initiation lead to higher mortality rates among HIV-infected children.^4^

Point-of-care (POC) testing for EID is a recent innovation that permits health care systems to decentralize testing and bypass the inefficient networks needed for centralized testing platforms.^3^ POC EID has well-documented impact and success, virtually eliminating turnaround times and therefore permitting a same-day testing and treatment paradigm for HIV-infected infants.^5^^–^^8^ In Mozambique, a cluster randomized trial showed that 90% of participants accessing POC EID were linked to timely ART compared with 13% in the standard of care.^9^

POC EID testing in Mozambique uses the Abbott mPIMA platform, which gives results in approximately 1 hour, and was scaled up from 2017 and 2018 in a strategic rollout that prioritized deployment to high-volume outpatient services at public primary health care facilities, consistent with international implementation recommendations.^10^^,^^11^ In recognition of the opportunity to also reach hospitalized infants who are more likely to have advanced HIV and need urgent ART to prevent mortality, pediatric wards in 2 large referral hospitals, Hospital Central de Maputo (HCM) and Hospital Central de Beira (HCB), were included in the implementation plan. Despite their perceived ease of access to reference laboratories with conventional molecular testing equipment, inpatient wards in hospitals in Mozambique historically face similar long turnaround times for EID results compared with outpatient health facilities, with an average of 36 days for the 11 largest hospitals in the country in 2016 compared with 47 days for health centers.^12^ These settings represent considerable wastage of conventional EID resources because patient discharge usually occurs before results are available, and post-discharge follow-up is complicated by long distances between patients' homes and the referral hospital. Furthermore, the national EID program does not use a unique patient identifier to facilitate tracking results across health facilities. As such, conventional EID programs do not adequately serve pediatric inpatient wards and represent a missed opportunity, especially since these settings are known to have high yields of HIV-positive tests when EID testing is part of provider-initiated testing and counseling (PITC) is operationalized.^13^^–^^17^

Conventional EID programs do not adequately serve pediatric inpatient wards and represent a missed opportunity to reduce the impact of HIV in children.

We conducted an early assessment of the impact of POC EID deployment in the pediatric wards of HCM and HCB.

## METHODS

### Study Design, Setting, and Participants

This study was a retrospective review of routine EID testing and patient care data from HCM and HCB, tier-4 reference hospitals providing the highest level of care in the public sector in the southern and central regions of Mozambique, respectively. In 2018, HCM admitted an average of 866 children per month, and HCB admitted an average 536 children per month. In both hospitals, the median age of admission is below 2 years old. The POC study period began from the time of POC implementation at each site (February 2017 for HCB and September 2017 for HCM) through July 2018. Eight months of pre-POC data, when testing was performed via dried blood spots (DBSs), were included from each site for comparison. Per national guidelines, HEIs aged 1 to 18 months who underwent inpatient EID testing were included.^18^ Patients with nonvalid EID results and those referred from other health facilities for EID testing (i.e., infants not admitted to the hospital but accessing the POC EID platform) were excluded. Patients who were known to be HIV infected at the time of admission were not eligible for EID testing and were not included.

### National PITC and EID Guidelines

Infants already known to be HIV exposed at the time of admission (identified through review of maternal and child health documents and caregiver medical history) were routinely offered initial EID testing if standard testing at 1 month of age had been missed. They were also generally offered repeat EID testing if they had outdated previous negative outpatient EID test results or presenting conditions suggestive of HIV infection. For infants whose mothers had unknown serostatus or whose last negative test was more than 3 months prior to admission, national policy was to conduct routine opt-out ward-based PITC of mothers using rapid antibody tests to newly identify HEIs eligible for EID testing.^18^ The national EID algorithm during the time period of this study called for virologic testing at enrollment in the HEI clinic (recommended at 1 month of age), at 9 months if rapid antibody testing was positive, and at any time infants had signs and symptoms suggestive of HIV infection. For inpatient EID, guidelines are not specific about when to repeat virologic testing for HEIs who previously tested negative, but generally speaking, EID testing is repeated in children with malnutrition, developmental delay, or infectious illness that could be associated with immune suppression, or if previous testing was more than 2 months prior to admission. Active phone tracing of infected patients identified through hospital-based EID to confirm their continuity of care after discharge to primary health centers was recommended, but it was more routinely done at HCM than HCB during the period of this study.

### Data Collection

For the pre-POC period, DBS EID data were collected for each site from a national EID data database that contains test results, demographic information, and clinical information from standard national EID requisition forms which include PMTCT information on maternal ART, infant prophylaxis, breastfeeding status, and previous EID testing. ART information for the pre-POC period was obtained from site ART registers. A more comprehensive set of data were collected for the POC EID period from sources including onsite EID logbooks, EID requisition forms, 2 web-based databases (POC connectivity and the national online EID portal), ART registers, and call logs from a patient follow-up program (HCM only). A trained team of data collectors reviewed all available data sources to populate an anonymous, structured database that recorded patient demographics, EID testing dates and results, ART information, and follow-up status for all patients with POC EID testing in the study period.

### Data Analysis

Data were collected and organized into Microsoft Excel (2003), and data analysis was conducted using STATA v12 (StataCorp©, 2011). Descriptive summary statistics were produced for testing volumes and positivity rates. Chi-square and Fisher's exact tests were applied to investigate differences in pre- and post-POC implementation results in addition to POC positivity rates for a set of patient characteristics including age, sex, PMTCT regimens, and previous access to conventional DNA polymerase chain reaction (PCR) results. All statistical analyses used *P*-values and 5% significance level for inference.

### Ethical Considerations

Ethical approval for this assessment was obtained from Mozambique's National Health Bioethics Committee reference 80/CNBS/14. The Scientific Directorates of HCM and HCB also approved the study. These boards did not require the study to obtain consent from caregivers for use of the routine EID and ART data included in the analysis.

## RESULTS

### POC Study Population Characteristics

A total of 511 HEIs were tested with POC at both hospitals during the study period (330 patients over 18 months at HCB, and 181 patients over 11 months at HCM). The median age was 5 months, and 232 patients (45.4%) were girls. A previous negative DNA PCR performed at the primary health facility level of care as part of routine EID was documented for 219 (42.9%) infants. No history of antiretroviral prophylaxis or ART for PMTCT was available for 136 (26.6%) of mothers, 146 (28.6%) of infants had not received nevirapine prophylaxis, and 326 (63.8%) of infants were still being breastfed (Table 1).

**TABLE 1.** Clinical and Demographic Characteristics of HIV-Exposed Infants in Point-of-Care Early Infant Diagnosis Study From Inpatient Wards of 2 Pediatric Referral Hospitals, Mozambique

Variable	Patients, *n* (%)		
	HCB	HCM	Total
Age, months			
1–2	101 (30.6)	45 (24.9)	146 (28.6)
3–5	67 (20.3)	54 (29.8)	121 (23.7)
6–8	73 (22.1)	33 (18.2)	106 (20.7)
9–11	40 (12.1)	27 (14.9)	67 (13.1)
≥12	48 (14.5)	20 (11.0)	68 (13.3)
Missing	1 (0.3)	2 (1.1)	3 (0.6)
Sex			
Female	148 (44.8)	84 (46.4)	232 (45.4)
Male	182 (55.2)	97 (53.6)	279 (54.6)
Previous DNA PCR			
Yes	138 (41.8)	81 (45.0)	219 (42.9)
No^a^	192 (58.2)	99 (55.0)	292 (57.1)
Maternal PMTCT			
Prophylaxis	2 (0.6)	4 (2.2)	6 (1.2)
Full ART	210 (63.6)	133 (73.5)	343 (67.1)
None	105 (31.8)	31 (17.1)	136 (26.6)
Missing	13 (3.9)	13 (7.2)	26 (5.1)
Infant PMTCT			
NVP prophylaxis	207 (62.7)	130 (71.8)	337 (65.9)
None	113 (34.2)	33 (18.2)	146 (28.6)
Missing	10 (3.0)	18 (9.9)	28 (5.5)
Current breastfeeding			
Yes	233 (70.6)	93 (51.4)	326 (63.8)
No	78 (23.6)	73 (40.3)	151 (29.5)
Missing	19 (5.8)	15 (8.3)	34 (6.7)
Total	330 (100)	180 (100)	511 (100)

Abbreviations: ART, antiretroviral therapy; HCB, Hospital Central de Beira; HCM, Hospital Central de Maputo; NVP, nevirapine; PCR, polymerase chain reaction; PMTCT, prevention of mother-to-child transmission.

a Includes infants with unknown or undocumented previous testing.

### POC Test Results and ART Initiation

POC tests were positive in 152 (29.7%) of the HEIs tested. Of these HIV positive infants, 74 (48.7%) initiated ART during their hospitalization. A total of 43 inpatient deaths (28.2%) occurred among infants with confirmed HIV infection, including 12 who initiated ART in the hospital. Excluding the 31 infants who died in the hospital prior to ART initiation, 61.2% (74/121) of eligible patients initiated ART while admitted. For the 109 infected infants who were discharged, 66.1% (41/62) of those who initiated ART as inpatients were confirmed to be active on ART on follow-up, and only 29.8% (14/47) of those who did not initiate ART as inpatients were confirmed to be active on ART (Figure).

**FIGURE:**
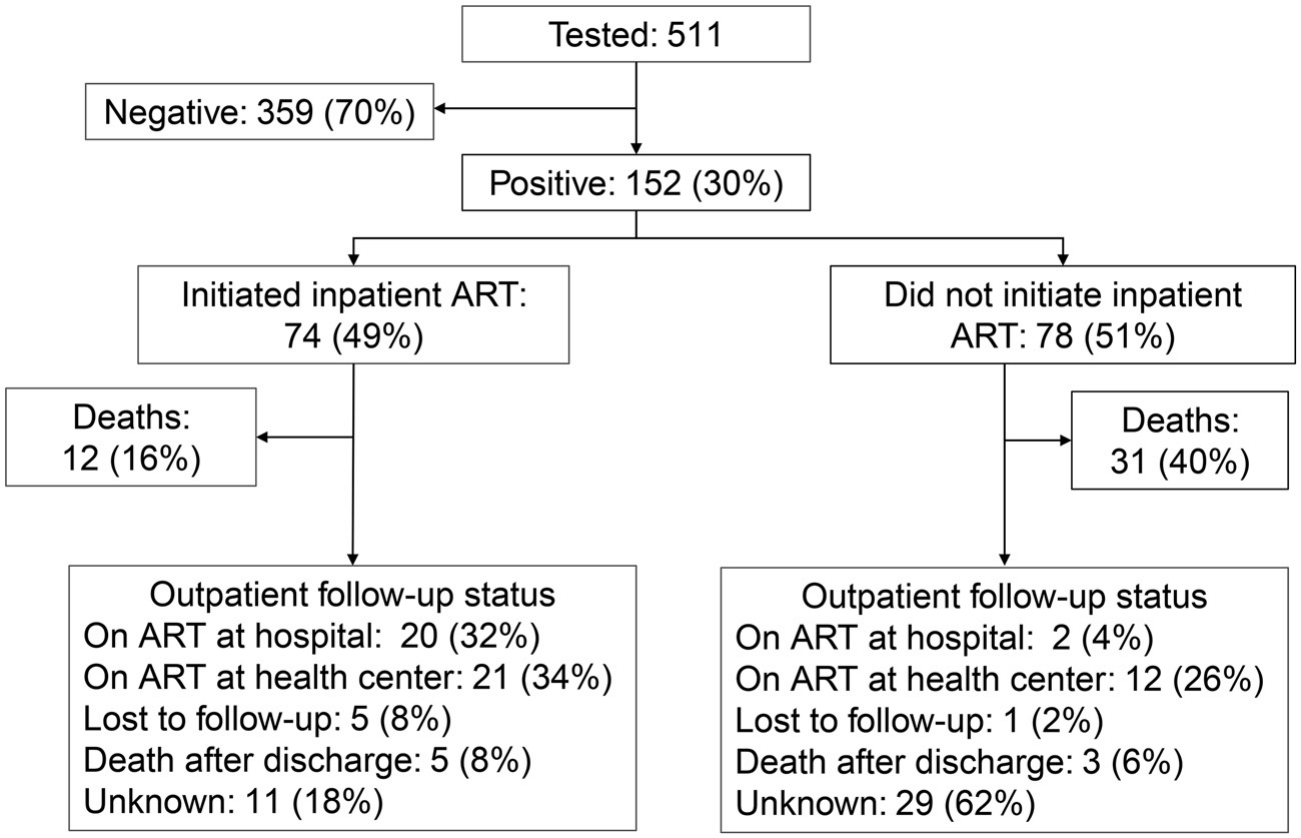
Point-of-Care Early Infant Diagnosis Testing and Linkage to Antiretroviral Therapy, Among HIV-Exposed Infants From Two Pediatric Referral Hospitals, Mozambique Abbreviation: ART, antiretroviral therapy.

### Clinical and Demographic Variables Associated With POC Positivity

Infants whose mothers were on ART had a 27.4% positivity rate compared with 38.2% when there was no maternal ART (*P*=.047). Infants who had received nevirapine prophylaxis had a 26.4% positivity rate compared to 37.0% in those who had not (*P*=.018). Infants who were still breastfeeding at the time of testing had a 33.1% positivity rate compared with 22.5% in those who were not (*P*=.019). No significant differences in positivity were found based on age, sex, and previous negative DNA PCR (Table 2). Of children who tested positive, 37.5% (52/152) had a documented previous negative DNA PCR test performed prior to admission.

**TABLE 2.** Point-of-Care Early Infant Diagnosis Positivity by Clinical and Demographic Variables Among HIV-Exposed Infants From Inpatient Wards of 2 Pediatric Referral Hospitals, Mozambique

Variable	Negative Test, *n* (%)	Positive Test, *n* (%)	Chi-Square *P* Value
Age, months			
1–2	113 (77.4)	33 (22.6)	0.127
3–5	77 (63.6)	44 (36.4)	
6–8	70 (66.0)	36 (34.0)	
9–11	48 (71.6)	19 (28.4)	
≥12	49 (72.1)	19 (27.9)	
Missing	2 (66.7)	1 (33.3)	
Sex			
Female	160 (69.0)	72 (31.0)	0.561
Male	199 (71.3)	80 (28.7)	
Previous DNA PCR			
Yes	162 (74.0)	57 (26.0)	0.106
No	196 (67.4)	95 (32.6)	
Maternal PMTCT			
Prophylaxis	5 (83.3)	1 (16.7)	0.047^a^
Full ART	249 (72.6)	94 (27.4)	
None	84 (61.8)	52 (38.2)	
Missing	21 (80.8)	5 (19.2)	
Infant PMTCT			
NVP prophylaxis	248 (73.6)	89 (26.4)	0.018
None	92 (63.0)	54 (37.0)	
Missing	19 (67.9)	9 (32.1)	
Current breastfeeding			
Yes	218 (66.9)	108 (33.1)	0.019
No	117 (77.5)	34 (22.5)	
Missing	24 (70.6)	10 (29.4)	
Total	359 (70.3)	152 (29.7)	

Abbreviations: ART, antiretroviral therapy; NVP, nevirapine; PCR, polymerase chain reaction; PMTCT, prevention of mother to child transmission.

a Fisher's exact test.

### POC Positivity by Ward

Significant differences in POC test positivity were found based on the ward from which patients were referred for inpatient testing, with malnutrition (41.1%), the pediatric intensive care unit (40.0%), and the breastfeeding ward (38.3%) having the highest rates (Table 3).

**TABLE 3.** Point-of-Care Early Infant Diagnosis Positivity Among HIV-Exposed Infants From Inpatient Wards of 2 Pediatric Referral Hospitals, Mozambique, by Referral Ward

Ward	Negative Test, *n* (%)	Positive Test, *n* (%)	Fisher's Exact Test *P* Value
Nursery	12 (100.0)	0 (0)	0.002
Breastfeeding^a^	108 (61.7)	67 (38.3)	
General ward^b^	32 (82.1)	7 (17.9)	
Infectious diseases	130 (74.7)	44 (25.3)	
Malnutrition	33 (58.9)	23 (41.1)	
Pediatric intensive care unit	15 (60.0)	10 (40.0)	
Other^c^	5 (83.3)	1 (16.7)	
Missing	24 (100.0)	0 (0)	
Total	359 (70.3)	152 (29.7)	

a Ward for all admission diagnoses in patients aged 1–12 months at Hospital Central de Maputo. During the study, Hospital Central de Beira changed admission criteria to breastfeeding ward from 1–6 months to 1–12 months.

b Patients aged 12 months or older.

c Includes pediatric surgery, respiratory, and other subspecialty wards.

### Utilization, Positivity, and Linkage to ART Compared With Pre-POC DBS Testing

In combined analysis from both hospitals, testing volume increased 97% from an average of 8.9 inpatient tests per month with DBSs in the pre-POC period to 17.6 tests per month with POC (*P*<.001). The median age of tested infants was 4 months for the pre-POC period and 5 months for the POC period. Test positivity decreased from 45.5% pre-POC to 29.7% with POC (*P*<.001). Documentation of successful linkage to ART (inpatient or outpatient) increased from 35.4% pre-POC to 57.9% with POC (*P*=.002).

## DISCUSSION

This study shows that inpatient POC EID is both feasible and effective. Not only did the placement of POC technology in these hospital settings increase overall testing volume and newly identify a high number of children with HIV-positive tests, but the immediate onsite POC test results also permitted much improved linkage to ART for these children compared with the pre-POC period.

The immediate onsite POC test results permitted much improved linkage to ART for children with HIV infection compared with the pre-POC period.

A highly significant 97% increase in testing volume per month occurred after allocation of POC, which we believe is best explained by clinicians being more likely to order EID testing when timely results were available. Improved inpatient PITC at both hospitals during the time period of the study could have contributed to more newly identified HEIs, but PITC testing data were not included in this study and the large majority of EID tests are performed in infants already known to be HIV exposed. No dramatic changes in maternal antenatal HIV prevalence, annual admission volumes, or EID eligibility criteria occurred during the time period of the study that would otherwise explain this large increase in testing volume. Yet, despite the large increase in testing volume, the POC platforms still may have been underutilized. This circumstance is of particular concern at HCM, which is in a higher-HIV prevalence province and has approximately 60% more admissions per month, but had fewer inpatient POC tests performed per month than did HCB (16.5 versus 18.3).^19^ Increased POC EID testing is needed in wards that admit HEIs 12–18 months of age, which represented only 36.6% of the test requests in this study. This finding is important in light of new EID algorithm recommendations from the World Health Organization that call for virologic testing in HEIs >9 months of age given the possibility of false-negative rapid HIV test results.^11^^,^^20^^–^^22^

Concerningly, a study of pediatric inpatient PITC from wards in Mozambique reported that coverage rates are low.^13^ Routine opt-out rapid antibody testing of breastfeeding mothers is a crucial first step to newly identify HEIs who need EID testing, and this step can help improve POC EID platform utilization. The same study also showed that hospitals that relied on DBSs for EID often did not test or retest exposed infants and presumptive HIV diagnosis was rarely made.^13^ POC platforms bypass the inherent delays that come with DBS-based EID; eliminate the need for inpatient presumptive HIV diagnosis, which has been consistently underutilized; and have the potential to strengthen pediatric inpatient PITC and improve case detection. Inpatient POC EID can also serve as an important backstop to traditional outpatient HEI-clinic EID, as 37.5% of the infants with positive POC tests had a previous negative outpatient PCR and were presumably infected during breastfeeding.

The 29.7% overall positivity rate for POC EID from this study is significantly higher than other results reported from the region. A POC EID evaluation from 8 African countries reported a 15.2% prevalence in pediatric inpatients (hospitalized children represented only 2.9% of the total cohort), and a study from Malawi, where 48.9% of the infants tested with POC came from inpatient wards, had an overall positivity rate of 5.7% (no inpatient/outpatient disaggregation provided).^5^^,^^7^

The EID positivity rate was even higher before POC implementation, at 45.5%. Although some of this difference could be explained by improved PMTCT coverage in the later POC period, it is also likely that DBS testing was only performed in HEIs with more advanced signs and symptoms of HIV infection.^1^ Not all admitted HEIs need repeat virologic testing with POC, but the ease of use and timely results seem to lower the clinical threshold for testing to include more subtle early signs of infection, thereby allowing for more timely diagnosis of HIV with better treatment outcomes.

The PMTCT program in Mozambique continues to struggle to reduce vertical transmission, with an estimated rate of 15% (much of which occurs via breastfeeding), challenges with maternal ART adherence and retention, and high rates of maternal seroconversion during pregnancy and lactation.^23^^–^^26^ Undiagnosed infants will often become ill and require hospitalization, and this study showed the high toll of such late presentation in terms of inpatient mortality at 28.2%. This rate was higher than the 22% mortality rate reported in a recent pediatric inpatient study from Kenya that included infants and older children, and it highlights the importance of strengthening serial maternal HIV testing throughout pregnancy and the breastfeeding period, as well as outpatient EID, so diagnoses can be made before children develop advanced disease requiring hospitalization.^27^

The PMTCT program in Mozambique struggles to reduce vertical transmission.

Confirmed ART initiation for infants with positive inpatient EID tests was 64% higher in the POC period, facilitated by same-day actionable results. Despite previous efforts to prioritize central laboratory testing of DBS samples from inpatient wards, the turnaround times for conventional DNA PCR (36-day average for the 11 largest hospitals in Mozambique in 2016) meant that most hospitalized patients had been discharged before their results were returned, despite being expedited.^12^ Furthermore, posthospitalization follow-up often occurs at primary health care centers closer to patients' homes, and the resources needed for routine active tracing of infected infants via phone or home visits are generally lacking. Consequently, these conventional results never reached the children's caregivers, contributing to reduced linkage to ART. This study shows that such hurdles to timely ART initiation for infants with HIV infection diagnosed during hospitalization can be addressed with the use of POC EID.

With the exclusion of infants who died during hospitalization prior to ART initiation, 61.2% of those with positive tests initiated as inpatients. Our data set did not allow for analysis of why some infants did not initiate ART as inpatients, but anecdotally the most common reason was inpatient tuberculosis diagnosis with guidelines recommending 2 weeks of treatment before ART should be started. An additional 29.8% of HIV-infected infants who were discharged without ART were confirmed to have initiated as outpatients. These numbers compare unfavorably to other POC studies, which reported ART linkage rates of 86.3% and 91%, but did not disaggregate inpatient versus outpatient timing.^5^^,^^7^ A large difference was found in confirmed ART post discharge in patients who initiated as inpatients versus those who did not (66.1% vs. 29.8%), suggesting that an ideal model would promote ART initiation during admission to the extent possible. A recent clinical trial showed no early mortality benefit to urgent inpatient ART initiation in children, but the findings from this study suggest that initiation after stabilization but before discharge may help improve access to and retention on ART.^27^ A clear need exists to improve active follow-up of infants diagnosed during hospitalization at these sites to ensure successful linkage to ongoing ART.

The results from this study suggest that POC EID can further improve access to EID and pediatric ART by broadening the considerations for potential placement scenarios. Our findings suggest there is clear benefit to deploying POC testing to hospitals with subsequent establishment of context-appropriate ART initiation models that readily decentralize back to primary care. However, this consideration for further deployment of POC testing to inpatient settings does not need to be limited to large pediatric hospitals; the dynamics of inpatient reality (late presentation, incomplete EID testing history, admission duration shorter than conventional DNA PCR turnaround times) are applicable to smaller wards throughout the country whose patients would also benefit from inpatient POC EID. The ability to now multiplex on POC instruments, such as the HIV viral load assays that are now also being performed at HCM and HCB on the Abbott mPIMA analyzer used in this study, the advent of birth EID testing opportunities, and combined inpatient/outpatient testing at sites with both EID clinics and pediatric wards can generate testing volumes that justify deployment to settings such as district hospitals that previously may not have met POC allocation criteria. Indeed, there remain deployment opportunities to further reach HEIs across the health care system, and POC consistently proves to be an effective and feasible solution—one that will be needed to help reach national and global targets.

There is clear benefit to deploying POC testing to hospitals with subsequent establishment of context-appropriate ART initiation models.

### Limitations

This study had limitations that need mention. A smaller set of data variables was available for the pre-POC period, and we were not able to perform a comparison of hospital outcomes from before and after the implementation of inpatient POC EID. The number of HIV-exposed children who were admitted and would need EID testing during the pre-POC and POC periods was also not part of the data available for this study, so our conclusions about availability of POC driving increased testing demand come with qualifications. Phone follow-up to verify outpatient ART status did not occur at a standard time interval post hospital discharge, and systems were lacking for timely and routine phone follow-up of discharged patients at HCB, possibly leading to an underestimation of ART linkage. Given the retrospective methodology, several clinical and demographic variables had missing data that could not be captured. The data presented here are from a 2017–2018 period, but the results reflect the inpatient EID realities in Mozambique and are useful for ongoing programmatic planning.

## CONCLUSIONS

Inpatient wards are a high-yield site for case identification and ART initiation that have historically been overlooked in PMTCT programming. POC platforms can increase inpatient EID testing volume and represent a transformative opportunity to improve the diagnosis and treatment of HIV in hospitalized infants. POC EID scale-up plans should include pediatric wards as priority sites for future expansion.

## Notes

**Peer Reviewed**

**First published online:** March 8, 2021.
